# Supplementary material for: What does it take to learn the rules of RNA base pairing? A lot less than you may think
Source: bioRxiv. 2025 Aug 2:2025.07.31.668042. Preprint. [Version 1] doi: 10.1101/2025.07.31.668042 (PMC12324431; doi:10.1101/2025.07.31.668042)
Supplement: Supplement 1 [file media-1.gz › supplemental_material/experiments/g6_optimize_param_S288C_chrV_YER019C-A.rm_v1.200_uniform/losses_i80.pdf]

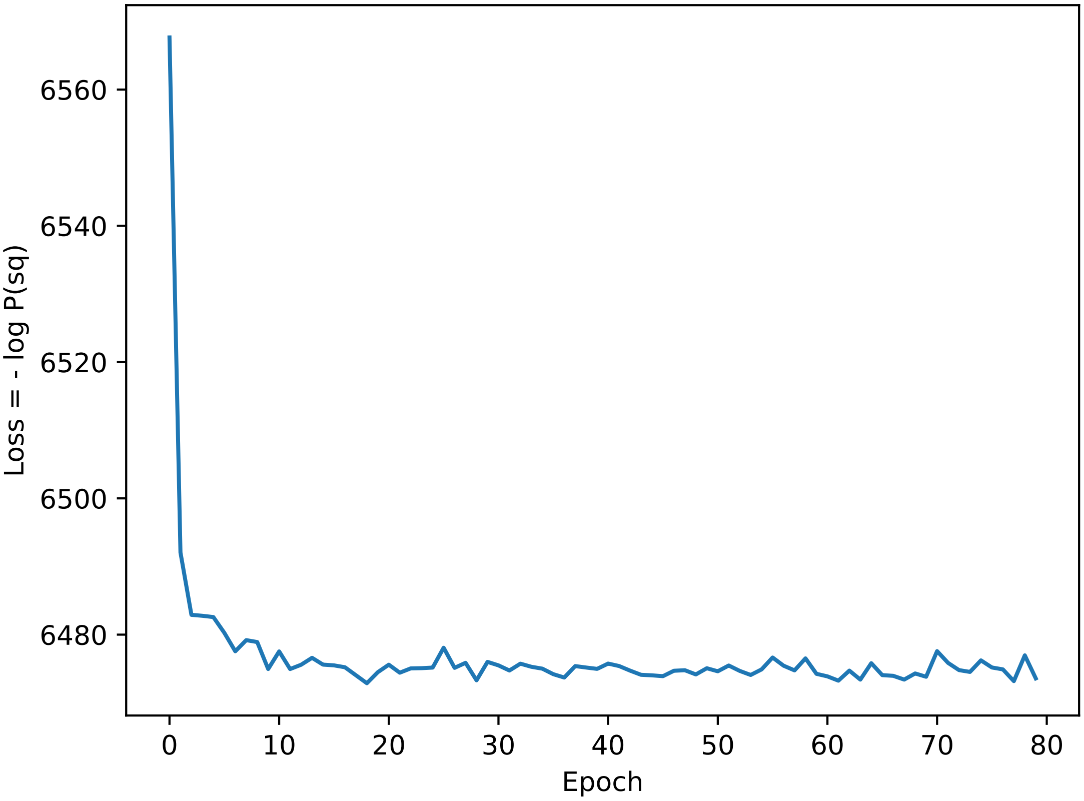

Loss = - log P(sq)
6560
6540
6520
6500
6480
0
10
20
30
40
50
60
70
80
Epoch
